# Death from the Inhalation of Ether

**Published:** 1877-12

**Authors:** 


Death from the Inhalation of Ether.—We regret to have to record a case in which the administration of ether terminated fatally, and which occurred in the practice of Dr. G. M. Lowe, of Lincoln. The patient was a lady forty-eight years of age, who had discharged the duties of a governess in the family of Dr. Lowe, and had for some time past been suffering from cancer of the breast. A consultation was held, and the removal of the tumor determined upon. The administration of the ether was confided to Dr. Mitchinson, who had had large experience in its employment. All proper precautions appear to have been taken. Dr. Mitchinson examined the heart, and, finding it rather feeble, directed the patient to take a little brandy and water. She was quite cheerful, though somewhat nervous. Half an ounce of ether was poured on the inhaler, which was placed over the mouth in the usual way. The valves were open, and gave free ingress and egress to the air. After a few inhalations the patient's face suddenly became turgid and the hands white. The inhaler was at once removed, and the tongue brought forward, cold water dashed over the face, and the chest rubbed with brandy, but the breathing became stertorious, the face more and more congested, the pulse failed, there was an effort at vomiting, and death took place within a few seconds.—*Lancet.*

Germs in Disease.—According to observations reported to the French Academy, MM. Pasteur and Joubert show that the terrible animal disease which is known as *charbon or sang de rate* (carbuncular gangrene), is caused by microscopic bacteridia, which was first observed by Dr. Davaigne, in 1850. It may, therefore, be classed with trichinosis and the itch, as a parasitic disorder. Vibrios, bacteria and bacteridia, are all found under two essentially distinct forms; either in translucent threads of variable length, multiplying rapidly by division, or in groups of little brilliant corpuscles formed in the interior of the threads, which separate from the parent, and constitute an apparently inert mass of points, from which countless legions of filiform individuals may come, having the same twofold methods of reproduction. The threads may be killed by drying, or by a heat much below that of boiling water. The germs, when dry, withstand temperature from 120 deg. to 130 deg. C., or 248 deg. to 266 deg. F.—*Medical and Surgical Reporter.*

